# Development of a Collision-Free Path Planning Method for a 6-DoF Orchard Harvesting Manipulator Using RGB-D Camera and Bi-RRT Algorithm

**DOI:** 10.3390/s24248113

**Published:** 2024-12-19

**Authors:** Zifu Liu, Rizky Mulya Sampurno, R. M. Rasika D. Abeyrathna, Victor Massaki Nakaguchi, Tofael Ahamed

**Affiliations:** 1 Graduate School of Science and Technology, University of Tsukuba, 1-1-1 Tennodai, Tsukuba 305-8577, Japan; 2 Department of Agricultural and Biosystem Engineering, Universitas Padjadjaran, Sumedang 45363, Indonesia; 3 Department of Agricultural Engineering, University of Peradeniya, Kandy 20400, Sri Lanka; 4 Institute of Life and Environmental Sciences, University of Tsukuba, 1-1-1 Tennodai, Tsukuba 305-8577, Japan

**Keywords:** path planning, Bi-RRT, collision-free, 6-DoF manipulator, harvesting robots

## Abstract

With the decreasing and aging agricultural workforce, fruit harvesting robots equipped with higher degrees of freedom (DoF) manipulators are seen as a promising solution for performing harvesting operations in unstructured and complex orchard environments. In such a complex environment, guiding the end-effector from its starting position to the target fruit while avoiding obstacles poses a significant challenge for path planning in automatic harvesting. However, existing studies often rely on manually constructed environmental map models and face limitations in planning efficiency and computational cost. Therefore, in this study, we introduced a collision-free path planning method for a 6-DoF orchard harvesting manipulator using an RGB-D camera and the Bi-RRT algorithm. First, by transforming the RGB-D camera’s point cloud data into collision geometries, we achieved 3D obstacle map reconstruction, allowing the harvesting robot to detect obstacles within its workspace. Second, by adopting the URDF format, we built the manipulator’s simulation model to be inserted with the reconstructed 3D obstacle map environment. Third, the Bi-RRT algorithm was introduced for path planning, which performs bidirectional expansion simultaneously from the start and targets configurations based on the principles of the RRT algorithm, thereby effectively shortening the time required to reach the target. Subsequently, a validation and comparison experiment were conducted in an artificial orchard. The experimental results validated our method, with the Bi-RRT algorithm achieving reliable collision-free path planning across all experimental sets. On average, it required just 0.806 s and generated 12.9 nodes per path, showing greater efficiency in path generation compared to the Sparse Bayesian Learning (SBL) algorithm, which required 0.870 s and generated 15.1 nodes per path. This method proved to be both effective and fast, providing meaningful guidance for implementing path planning for a 6-DoF manipulator in orchard harvesting tasks.

## 1. Introduction

With the decreasing and aging agricultural workforce, fruit harvesting, a labor-intensive task, is facing a shortage of labor [1,2]. In this situation, fruit harvesting robots equipped with high degrees-of-freedom (DoF) manipulators are seen as a promising solution for performing harvesting operations in conventional and unstructured orchards [3,4]. However, the deployment of such robots involves various technical challenges [5,6,7]. Path planning is one of the most critical technical challenges, as it enables robotic manipulators to navigate within canopies, thereby achieving efficient apple harvesting [8,9,10,11,12]. The path planning process integrates data from sensors, such as depth cameras or laser sensors, to accurately perceive the environment and determine a collision-free path, guiding the manipulator from its starting position to the target fruit while effectively avoiding obstacles [13,14,15]. Efficient path planning not only ensures the robot’s safe operation within the orchard but also directly affects the overall efficiency and success rate of fruit harvesting operations [16,17].

In recent years, to enhance harvesting efficiency, many studies have focused on developing effective path planning for a fruit harvesting robotic system in orchards [18,19,20]. Tang et al. (2021) proposed an Improved Artificial Potential Field (IAPF) algorithm to address local minimum traps and target not reachable problems in path planning for a 6-DoF manipulator. The IAPF achieves a 54.89% reduction in computation duration and lowers aggregate joint deviation by 45.41% compared to Artificial Potential Field (APF), with higher planning efficiency and real-time performance in citrus-picking tasks [21]. Liu et al. (2022) developed a Time-Optimal RRT (TO-RRT) algorithm for citrus-picking manipulators, aiming to enhance path-planning speed and obstacle avoidance. Their algorithm incorporates potential fields and a “step-size dichotomy” method, which improves the random tree’s directional search ability. Compared to conventional RRT algorithms, TO-RRT achieved significantly faster planning times and reduced path lengths in both simulation and real-world environments, optimizing the performance for picking tasks [22]. Yan et al. (2024) introduced an improved Ant Colony Optimization (ACO) algorithm for 3D path planning in apple orchards, designed specifically for obstacle avoidance. They integrated a biomimetic optimization mechanism to enhance search stability and used a B-spline for path smoothing. Moreover, the algorithm was tested on different apple tree shapes and showed high success rates and faster planning times, making it a promising solution for avoiding obstacles during apple harvesting [23].

Despite these studies, achieving both practical implementations and effective collision-free in path planning for real-world scenarios remains a major challenge. On the one hand, many existing studies rely on manually constructed environment map models. However, in practical scenarios, path planning requires the detection of the actual environment using depth sensors, with the detected data serving as the foundation for path planning of the manipulator. On the other hand, although the existing methods demonstrate effectiveness in certain contexts, they often face limitations in planning efficiency and computational cost. Furthermore, methods tailored specifically for higher-DoF manipulators operating in orchard environments are still underdeveloped.

In response to these challenges, we introduced a collision-free path planning method specifically designed for a 6-DoF fruit harvesting robot. With the introduced method, spatial data captured by an RGB-D camera enabled the reconstruction of a 3D obstacle map upon which the Bi-RRT algorithm was introduced for path planning, offering a solution better suited to practical applications. Moreover, the introduced Bi-RRT algorithm establishes a favorable trade-off between simplicity and efficiency, positioning it well for use under computational constraints. These features address some of the shortcomings of the existing methods and provide a practical strategy for robotic harvesting tasks in unstructured orchard environments.

## 2. Methodology

### 2.1. Robotic System Overview

**Figure 1:**
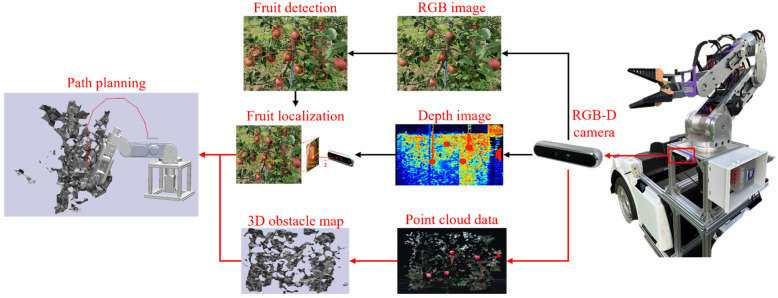
The process of manipulator path planning.

The fruit harvesting robotic system employed for this study featured a 6-DoF manipulator as its core component, as shown in Figure 1. This manipulator, designed to be cost-effective, included six revolute joints powered by closed-loop stepper motors paired with planetary reducers, enabling precise and complex fruit picking actions within orchards. The vision system was equipped with a RealSenseTM D455 RGB-D camera (Intel Corporation, Santa Clara, CA, USA), which acquires RGB and depth imagery to enable fruit detection and localization and 3D obstacle map reconstruction. The process of path planning began with the RGB-D camera capturing RGB image data to detect fruit using a deep learning model, identifying its position within the 2D image. Based on this 2D location, the depth data were then used to calculate the fruit’s 3D coordinates. The 3D coordinates were processed through inverse kinematics to determine the goal configuration for the end-effector. With this goal configuration established, and considering the reconstructed obstacle map, path planning algorithms were employed to establish a collision-free trajectory connecting the initial and goal configurations, enabling precise and efficient picking actions.

### 2.2. Fruit Detection and Localization

As a prerequisite for path planning, fruit detection and localization are essential steps to obtain precise fruit positions, enabling harvesting with a robotic system. The process begins with detecting fruits, identifying their bounding boxes, and determining their 2D positions in images [24]. For this step, we used the Faster-YOLO-AP detection algorithm from our previous work [25], a lightweight and efficient model built upon YOLOv8, adapted for real-time detection on low-power edge computing devices. The key innovations include the development of a smaller network, YOLOv8pico, by adjusting YOLOv8’s scaling factors; replacing the C2F feature extraction module with the PDWFasterNet module, incorporating partial depthwise convolution; employing depthwise separable convolution for downsampling to further reduce model size; and using EIoU loss for improved detection accuracy. On our apple dataset, Faster-YOLO-AP achieved an mAP@0.50:0.95 of 84.12% and had a faster inference speed. After detection, in order to achieve effective path planning, fruit localization calculates the 3D coordinates of the detected target by integrating its 2D position with depth data obtained by an RGB-D camera.

### 2.3. Determination of Manipulator’s Goal Configuration

After obtaining the 3D coordinates of the fruit’s position, our goal was to move the manipulator’s end-effector toward the corresponding fruit location. To achieve this, we needed to compute the goal joint angle configuration. This process involves establishing the kinematic model of the manipulator and selecting an appropriate inverse kinematics solution method. In this study, we first constructed the Denavit–Hartenberg (D–H) model of the manipulator and then employed the Levenberg–Marquardt (L–M) algorithm to solve the inverse kinematics, thereby determining the goal configurations.

#### 2.3.1. Kinematic Model of Manipulator

Accurate control of a manipulator’s movement relies on a clear understanding of its kinematic behavior. In general, this involves two fundamental processes: forward kinematics (FK), which computes the end-effector’s position and orientation from known joint angles, and inverse kinematics (IK), which determines the joint angles required to achieve a specified end-effector pose [26]. To perform these calculations of IK, a structured mathematical representation of the manipulator’s geometry is essential. Here, we employ the Denavit–Hartenberg (D–H) parameterization to describe the spatial relationships among the manipulator’s links. By systematically defining each link length $a_{i}$, link twist $\alpha_{i}$, link offset $d_{i}$, and joint angle $\theta_{i}$, the D-H method provides a consistent and manageable framework for both FK and IK solutions, enabling precise determination of the manipulator’s configuration [27].

#### 2.3.2. Inverse Kinematics of Manipulator

The IK problem for a manipulator involves determining the joint angles that will position the end-effector configuration at a desired pose, defined by its position and orientation [28]. Due to the non-linear and complex nature of the IK problem, especially for robotic manipulators with multiple degrees of freedom, analytical solution methods are typically difficult to obtain. Consequently, we employed the Levenberg–Marquardt (L-M) algorithm [29], which is a numerical solution method, to iteratively approximate the goal joint angles that reduce the discrepancy separating the current end-effector configuration from its target configuration. The L-M algorithm is particularly well-suited for addressing non-linear least squares tasks, as it blends gradient-based descent and Gauss–Newton methods. This combination enhances the algorithm’s robustness and convergence properties, making it effective for handling the intricacies of manipulator IK. Specifically, the aim of the L-M algorithm is to reduce the discrepancy, quantified by an error function, which measures the difference between the intended end-effector configuration and the one actually realized with the current joint angles:

(1) $$E\left(\theta \right)=\frac{1}{2}{\left\|T_{desired}-T_{\theta }\right\|}^{2}$$

where $T_{desired}$ is the homogeneous transformation matrix representing the target pose; $T_{\theta }$ is the transformation matrix derived from the current joint angles θ by calculating forward kinematics; and ‖·‖ denotes the Frobenius norm, measuring the difference between the two transformation matrices. At each iteration k, the L-M algorithm updates the joint angles θ using the following update rule:

(2) $$\theta_{k+1}=\theta_{k}-{\left(J^{T}J+\lambda I\right)}^{-1}J^{T}e$$

where J denotes the Jacobian matrix, a set of partial derivatives expressing how variations in joint angles influence end-effector pose; $e=T\left(\theta_{k}\right)-T_{desired}$ is the error vector at iteration k; λ is the damping factor that adjusts the influence between the gradient descent and Gauss–Newton methods; and I is the identity matrix.

### 2.4. Three-Dimensional Obstacle Map Reconstruction

**Figure 2:**
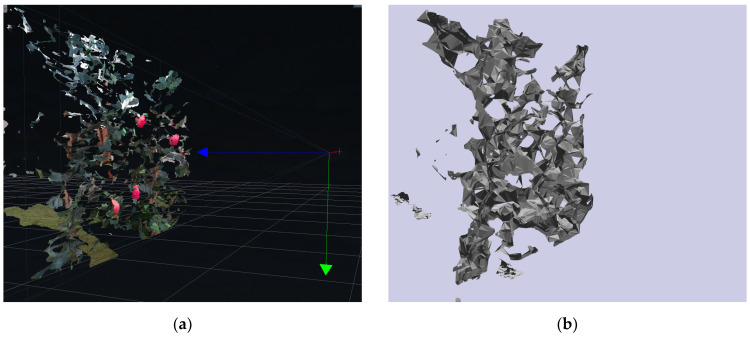
(**a**) Visualization of point cloud data. (**b**) Visualization of generated collision geometry.

The harvesting robot utilizes an Intel RealSense D455 RGB-D camera as an environmental sensor to detect and perceive obstacles within the workspace. The D455 camera captures both depth and color information, generating a comprehensive point cloud representation of the environment. The collected raw point cloud data consists of a set of three-dimensional points in the camera’s coordinate system, with each point containing spatial coordinates and color information, which is visualized as shown in Figure 2a. To integrate the obstacle information into the path planning framework of the manipulator, the raw point cloud data must be converted into a suitable format. Specifically, the data were processed to generate a collision geometry that the planning algorithms can utilize. This involves some preprocessing steps such as downsampling to reduce data density and removing statistical outliers to eliminate noise. Subsequently, a mesh of obstacles was constructed using techniques of alpha shapes and convex hulls. As shown in Figure 2b, a 3D obstacle map observed by the RGB-D camera was finally reconstructed and can be used for path planning.

Since the camera’s coordinate system differs from the robot’s global coordinate system, transformation is needed to transform the obstacle coordinates into the global coordinate system of the robot. The transformation involves applying a translation vector t and a rotation matrix R to the obstacle point cloud coordinates, formulated as follows:

(3) $$\left[\begin{array}{c} x_{glb}\\ y_{glb}\\ z_{glb} \end{array}\right]=R\left[\begin{array}{c} x_{cam}\\ y_{cam}\\ z_{cam} \end{array}\right]+t$$

where $\left(x_{cam},y_{cam},z_{cam}\right)$ represent the point cloud coordinates in the camera coordinate system, while $\left(x_{glb},y_{glb},z_{glb}\right)$ represent the transformed coordinates in the global coordinate system. This transformation ensures that the perceived obstacles are accurately positioned relative to the robot’s global coordinate system.

### 2.5. Simulation Model of the Manipulator

**Figure 3:**
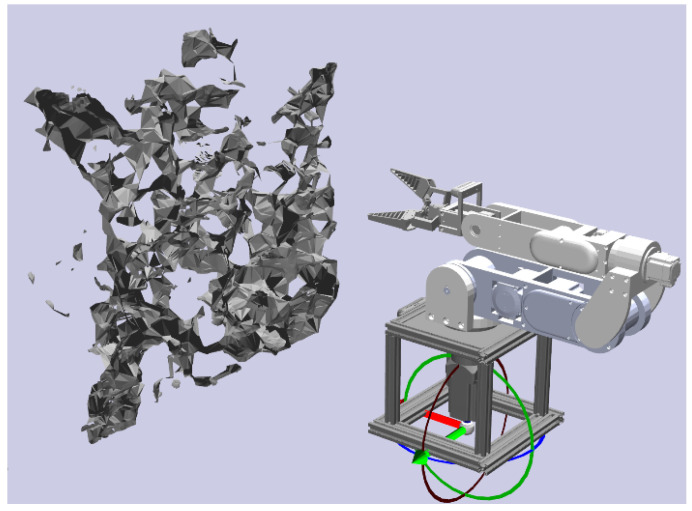
Visualization of the manipulator simulation model.

Next, we needed to build a simulation model for the manipulator. To standardize manipulator descriptions and facilitate interoperability among various software tools, we adopted the Unified Robot Description Format (URDF). The URDF offers a standardized file format that encapsulates multiple aspects of a manipulator’s physical configuration, including its links, joints, visual representation, and collision properties. We utilized a URDF export plugin to convert the manipulator model into a URDF file. The URDF file contains the necessary information for integration into the simulation environment and for collision detection with obstacles and the manipulator. As shown in Figure 3, we subsequently inserted the simulation model of the manipulator built by adopting the URDF file into the reconstructed 3D obstacle map.

### 2.6. Path Planning Algorithm

#### 2.6.1. RRT Algorithm

**Figure 4:**
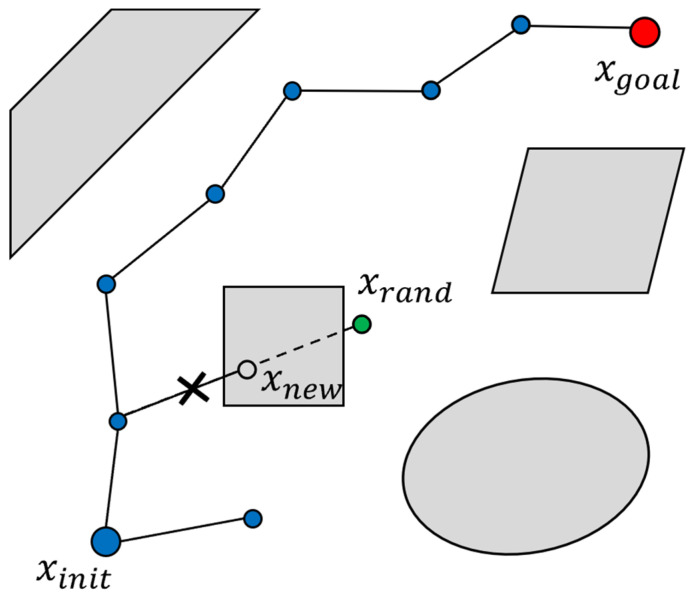
RRT expansion process.

Rapidly exploring random tree (RRT) is a path planning algorithm used to navigate high-dimensional configuration spaces [30]. As shown in Figure 4, the algorithm begins with an initial configuration $x_{init}$ and incrementally builds a tree through random sampling points $x_{rand}$ within the free space. For each sampled point, RRT identifies the nearest node $x_{nearest}$ within the existing tree T and then steers from $x_{nearest}$ toward the sampled point $x_{rand}$, generating a new node $x_{new}$ with a predefined *StepSize*. The algorithm will ensure that the path from $x_{nearest}$ to $x_{new}$ is collision-free before adding $x_{new}$ to the tree T. This process is repeated iteratively until a node is sufficiently close to the goal configuration $x_{goal}$ and a collision-free path to $x_{goal}$ is established. The procedure stops once an acceptable path connecting $x_{init}$ and $x_{goal}$ is found or the iteration limit $k_{max}$ is met. RRT explores the configuration space step by step by growing the tree toward randomly chosen points. This approach helps find feasible paths, even in environments with complex obstacles. Algorithm 1 presents the incremental tree-building process employed by RRT, utilizing random sampling and collision-free extension.
**Algorithm 1**: RRT Algorithm1:**Input:** $x_{init}$, $x_{goal}$, $k_{max}$2:**Output:** path from $x_{init}$ to $x_{goal}$3:**Initialization:** tree $T\leftarrow x_{init}$4:**For** each iteration from 1 to $k_{max}$
**do**5:  Sample $x_{rand}$ from the free space6:   Find $x_{nearest}$ in T closest to $x_{rand}$7:  Compute $x_{new}=Steer(x_{nearest},\;x_{rand},StepSize)$8:  **if** $CollisionFree(x_{nearest},\;x_{new})$
**then**10:   Add $x_{new}$ to T with parent $x_{nearest}$11:   **if** $\left\|x_{new}-x_{goal}\right\|<StepSize$
**then**12:   
**if**
$\;CollisionFree(x_{new},\;x_{goal})$
**then**
13:     Add $x_{goal}$ to T with parent $x_{new}$14:     **Return** path from $x_{init}$ to $x_{goal}$15:**end for**

#### 2.6.2. Bi-RRT Algorithm

**Figure 5:**
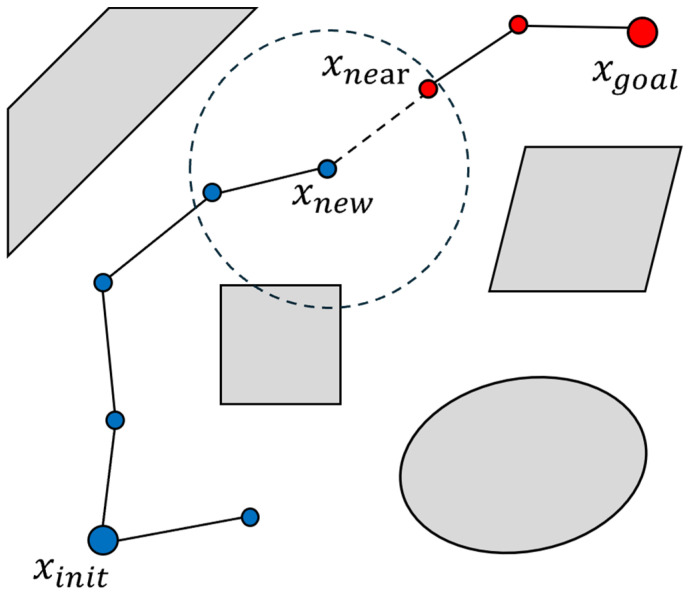
Bi-RRT expansion process.

To enhance the processing speed of the RRT procedure in high-dimensional configuration spaces, a bidirectional approach, bidirectional RRT (Bi-RRT), was proposed [31]. Instead of growing a single tree from the initial configuration $x_{init}$, Bi-RRT simultaneously grows two trees: one rooted at $x_{init}$ and the other at the goal configuration $x_{goal}$. This method enhances exploration efficiency by allowing the trees to search from both ends and increasing the likelihood of meeting in the middle, thereby reducing the time required to find an optimal path. In the Bi-RRT algorithm, both trees independently perform the standard RRT expansion and detection procedures using the same symbols and functions as previously defined in RRT, as shown in Figure 5. The bidirectional aspect involves alternating the expansion between the two trees and attempting to connect them whenever a newly generated node becomes available. Specifically, when a new additional node $x_{new}$ is integrated into the active tree $T_{a}$, the algorithm will calculate the nearest node $x_{near}$ within the other tree $T_{b}$. If $x_{new}$ and $x_{near}$ are within the connection distance d and the path between them is collision-free, the two trees are connected. As shown in Algorithm 2, the bidirectional implementation focuses on coordinating the growth of the two trees and connecting them when possible.
**Algorithm 2:** Bidirectional RRT* (Bidirectional Part Only)1:**Input:** $x_{init}$, $x_{goal}$, $k_{max}$2:**Output:** path from $x_{init}$ to $x_{goal}$3:**Initialization:** tree $T_{init}\leftarrow x_{init}$, $T_{goal}\leftarrow x_{goal}$4:**For** each iteration from 1 to $k_{max}$
**do**5: **if** iteration is even **then**6:  Set active tree $T_{a}=T_{init}$, the other tree $T_{b}=T_{goal}$7: **else**
8:  Set active tree $T_{a}=T_{goal}$, the other tree $T_{a}=T_{init}$9: Sample $x_{rand}$ from the free space10: Perform RRT expansion on $T_{a}$ using $x_{rand}$ to obtain $x_{new}$11:  This includes finding $x_{nearest}$, steering to $x_{new}$, and rewiring within $T_{a}$12: **if** $x_{new}$ is added to $T_{a}$
**then**13:  Find $x_{near}$ in $T_{b}$ closest to $x_{new}$14:  **if** $\left\|x_{new}-x_{near}\right\|<d$ and $CollisionFree(x_{new},\;x_{near})$
**then**15:   Connect $x_{new}$ and $x_{near}$ by adding edges in both trees16:   **Return** path from $x_{init}$ to $x_{goal}$ through $T_{a}$ and $T_{b}$17: Compute $x_{new}=Steer(x_{nearest},\;x_{rand},StepSize)$18:**end for**

## 3. Results and Discussion

### 3.1. Experimental Setup and Evaluation Metrics

**Figure 6:**
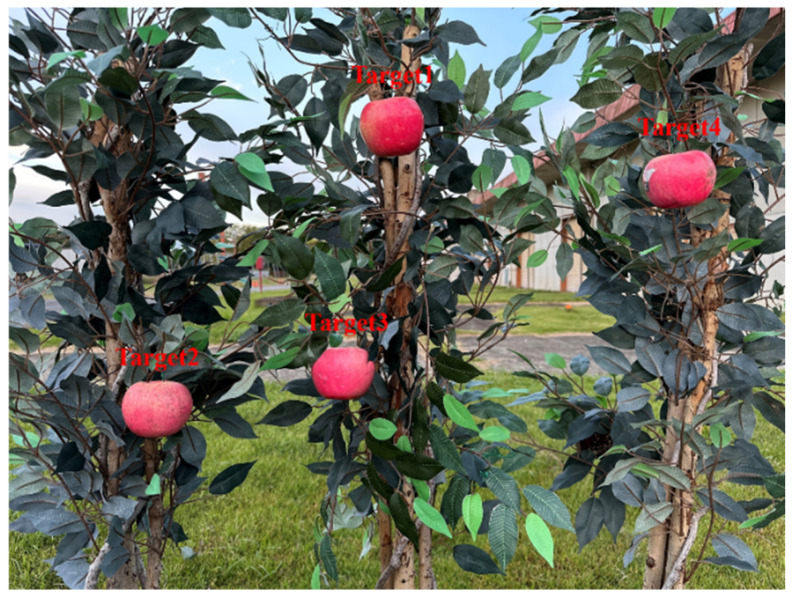
The artificial orchard environment.

To validate algorithm performance, we constructed an experimental setup simulating an orchard environment, featuring artificial trees and apples, as shown in Figure 6. In addition, all objects in the manipulator’s workspace may affect the path planning process, which means that any object in the workspace can be considered a potential obstacle. Therefore, we did not deliberately place obstacles directly in front of the fruit in the artificial orchard environment. Subsequently, using the previously described 3D obstacle map reconstruction method, a 3D obstacle map of the artificial orchard was generated. All subsequent experiments were conducted within this reconstructed map, ensuring consistency in testing.

The experimental code was executed on a local machine powered by a Core™ i5-12500 CPU (Intel Corporation, Santa Clara, CA, USA) and equipped with 16 GB of RAM. In experiments, the Klampt library managed the path planning process, RealSense SDK was utilized for depth data acquisition, and Open3D handled point cloud processing and the generation of collision geometries. The Klampt visualization module was used to display the manipulator, collision geometries, and the generated end-effector trajectories. The evaluation metrics in this study, including planning time and the number of path nodes, are widely selected in path planning research and provide a comprehensive assessment of the algorithm’s performance. Path planning time reflects the algorithm’s computational efficiency, which is critical for real-time operations in fruit harvesting robots. The number of path nodes represents path complexity, with fewer nodes typically indicating more efficient and direct paths, thereby enhancing operational efficiency.

### 3.2. Validation Experiment of Bi-RRT Algorithm

**Figure 7:**
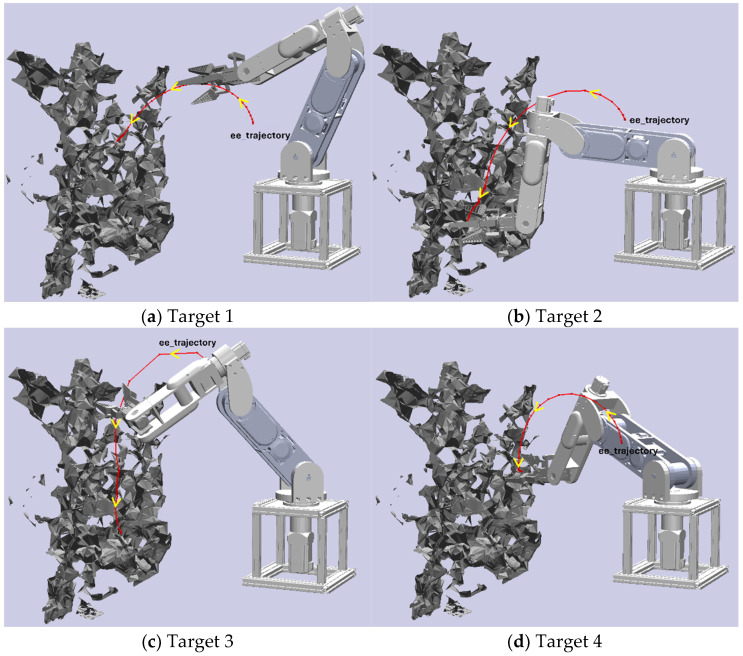
Picking trajectories of end-effector for different targets.

In the previous section, we introduced a collision-free path planning method with a 6-DoF manipulator for orchard harvesting based on the RGB-D camera and the Bi-RRT algorithm. To evaluate this algorithm’s effectiveness, we conducted four sets of experiments using four target apples within the artificial orchard environment. In each set, the Bi-RRT algorithm executed five tests to generate a trajectory leading to the target apple while avoiding obstacles. For each test, we recorded the planning time for the algorithm to generate a trajectory and the number of path nodes generated during the path planning process. To provide visual context, each experimental set is accompanied by a trajectory illustration, showcasing how the end-effector moves from its initial configuration toward its goal configuration, as illustrated in Figure 7.

The planning time and number of nodes demonstrated variability across different targets, as shown in Table 1. For Target 1, the planning times range from 0.762 to 0.989 s, with the number of path nodes varying between 6 and 11. For Target 2, planning times range from 0.496 to 1.809 s, while the number of path nodes is higher, ranging from 10 to 18. Target 3 exhibits planning times between 0.492 and 1.376 s, with the number of path nodes varying from 13 to 18, indicating increased complexity compared to other targets. One test in Target 4, in contrast, shows the shortest planning time of 0.368 s, with the number of path nodes of 25, suggesting uncertainty between time efficiency and path complexity. Overall, the Bi-RRT algorithm performed well across the four sets of experiments, demonstrating reliable path planning with a reasonable time and node generation, adapting effectively to the varying target positions.

### 3.3. Comparison Experiment with SBL Algorithm

To evaluate the efficiency of the introduced Bi-RRT, we conducted a comparison experiment with the Single-Query Bidirectional Lazy (SBL) path planning algorithm. The SBL algorithm minimizes collision checks by only validating paths when necessary [32]. As with Bi-RRT, four sets of experiments were conducted with SBL, with us performing five tests for each. Then, we calculated the average planning time and average number of nodes for each set to evaluate the performance of each algorithm.

Table 2 presents the average planning time and number of nodes for each experimental set with both algorithms. The Bi-RRT algorithm demonstrated an overall average planning time of 0.806 s across all sets, slightly outperforming SBL’s average time of 0.870 s. In terms of number of nodes, Bi-RRT produced an average of 12.9 nodes per path, which was fewer than the 15.1 nodes required by SBL. This suggests that Bi-RRT could potentially produce more direct paths to the target, enhancing planning efficiency. For specific sets, Bi-RRT consistently showed a slight advantage in average planning time for Target 1 (0.833 s), Target 3 (0.877 s), and Target 4 (0.649 s), whereas SBL performed more quickly for Target 2 (0.778 s). Meanwhile, Bi-RRT had fewer path nodes than SBL for all sets. These results indicate that Bi-RRT may be more effective in navigating toward certain targets within the artificial orchard environment, achieving efficient path planning with fewer required nodes.

### 3.4. Discussion

The experimental findings confirm Bi-RRT’s capability in directing the harvesting manipulator toward target positions. Across the four sets of experiments, the algorithm successfully generated a collision-free path, showing adaptability to the varying goal configurations of each target. Compared to the SBL algorithm, which required 0.870 s and generated 15.1 nodes per path on average, the Bi-RRT algorithm required just 0.806 s and generated 12.9 nodes per path, showed greater efficiency in path generation. In this study, environmental point cloud data were collected using an RGB-D camera and subsequently utilized to reconstruct a 3D obstacle map. A manipulator simulation model, built using a URDF file, was integrated into this reconstructed 3D map to perform path planning simulation experiments. As both the 3D obstacle map and the manipulator simulation model were derived from real-world data, the simulation experiments produced results that closely align with real-world conditions. More importantly, the simulation provided more detailed data, facilitating a more in-depth analysis of the manipulator’s path planning performance. Ultimately, our method was proven to be feasible and effective, meaning we propose a solid foundation for future practical applications.

The bidirectional expansion strategy effectively shortens the path planning time, reduces the search space, and increases the probability of discovering feasible paths by simultaneously expanding from both the start and goal points. This makes it particularly suitable for path planning in high-dimensional and complex environments. Both Bi-RRT and SBL belong to the category of bidirectional expansion algorithms, which is the reason why these two were chosen for comparison. Additionally, we have tried several other classic algorithms, such as PRM and RRT. However, these algorithms were unsatisfactory. For example, they only succeeded in finding a path two to three times out of ten attempts, and the quality of the generated paths was often poor, which made it impossible to compare effectively with them. We also recognize that Bi-RRT, as a well-established algorithm, has seen significant enhancements in recent years. While this study focused on the application of Bi-RRT in the context of orchard harvesting, we suggest that future work should integrate more advanced features and evaluate their performance in a dense orchard. In addition, the recent development of artificial intelligence networks offers new possibilities for path planning. In particular, reinforcement learning has shown potential in robotic path planning, but it typically requires a long training time [33,34]. As computational capabilities continue to improve, incorporating reinforcement learning could become a viable option for path planning in complex orchard environments.

We acknowledge that the current method has some limitations. In an orchard, obstacles can generally be classified into two types: soft obstacles, such as leaves, which can often be brushed aside, and hard obstacles, like branches, which must be fully avoided. Although this study recognizes the importance of classifying these obstacles, it has not yet adopted a specific method. Future work could focus on integrating deep-learning-based obstacle classification methods into the path planning process, such as using YOLO or Mask-RCNN segmentation algorithms to identify pixels of different obstacles in the image, and then mapping these pixels into the point cloud, removing the point cloud of soft obstacles and retaining only hard obstacles. Correspondingly, further experiments could be designed to verify the effectiveness of these methods in artificial and real orchard environments.

## 4. Conclusions

Effective path planning is essential for enabling autonomous navigation for high-DoF manipulators of harvesting robots within unstructured orchard environments. This process includes determining an efficient, collision-free trajectory that guides the robotic manipulator to pick the target fruit while avoiding obstacles. Although existing studies have made some progress, there are still limitations in relying on manually constructed obstacle map models or limitations are often faced in planning efficiency and computational cost. Therefore, in this study, we introduced a collision-free path planning method with a 6-DoF manipulator for orchard harvesting using an RGB-D camera and the Bi-RRT algorithm. Our primary contributions can be outlined as follows:

(1) **3D Obstacle Map Reconstruction:** By converting an RGB-D camera’s point cloud data into collision geometries, we implemented 3D obstacle map reconstruction, enabling the harvesting robot to perceive obstacles within its workspace.
(2) **Bi-RRT Path Planning Algorithm:** Implementing Bi-RRT enhanced overall efficiency and reliability. By simultaneously expanding in two directions from both the initial and goal configurations, Bi-RRT markedly reduced the path planning time and decreased the number of nodes in guiding the manipulator toward the target fruits.
(3) **Experiments:** The validation experiment and comparison experiment were conducted in an artificial orchard environment with multiple artificial trees and apples to assess the algorithms’ performance. In these experiments, we recorded evaluation metrics, including the planning time and number of path nodes, to thoroughly assess the algorithm’s efficiency in producing feasible, collision-free motion trajectories.
(4) **Results:** The experimental results demonstrated our method’s effectiveness, with the Bi-RRT algorithm achieving efficient collision-free path planning across all test sets. The average planning time per path was 0.806 s across all target sets, and the algorithm generated an average of 12.9 nodes per path. This demonstrates its superior path generation performance compared to SBL, which had an average planning time of 0.870 s and a number of path nodes of 15.1.

Therefore, the developed collision-free path planning method using an RGB-D camera and the Bi-RRT algorithm demonstrated both efficiency and effectiveness, providing a valuable guideline for implementing path planning in orchard harvesting tasks with a 6-DoF manipulator.

## Figures and Tables

**Table 1:** Results of the validation experiment.

	Goal Configuration	Planning Time (s)	Number of Path Nodes
Target 1	(0.04, −0.92, 0.14, 0.00, −1.04, 0.00)	0.762	6
		0.989	9
		0.840	7
		0.827	11
		0.873	10
Target 2	(−0.26, −1.48, −0.04, 0.00, −1.36, −0.42)	0.709	11
		0.698	10
		1.809	12
		0.615	16
		0.496	18
Target 3	(−0.34, −1.56, −0.36, 1.16, −1.38, 3.56)	0.910	15
		0.775	18
		1.376	13
		0.831	14
		0.492	17
Target 4	(0.42, −1.16, −0.14, 0.00, −0.98, 0.14)	0.368	25
		0.619	12
		0.969	10
		0.619	11
		0.671	12

**Table 2:** Results of the comparison experiment.

Algorithm	Experimental Set	Average Planning Time (s)	Average Number of Path Nodes
Bi-RRT	Target 1	0.833	8.6
	Target 2	0.865	13.4
	Target 3	0.877	15.4
	Target 4	0.649	14.0
	Total	0.806	12.9
SBL	Target 1	0.903	9.2
	Target 2	0.778	15.2
	Target 3	0.974	19.0
	Target 4	0.823	16.8
	Total	0.870	15.1

## Data Availability

The datasets generated and analyzed during this study are available from the corresponding author upon reasonable request, but restrictions apply to the data reproducibility and commercially confident details.
